# Long-Term Passenger Flow Forecasting for Rail Transit Based on Complex Networks and Informer

**DOI:** 10.3390/s24216894

**Published:** 2024-10-27

**Authors:** Dekui Li, Shubo Du, Yuru Hou

**Affiliations:** 1 College of Computer Science, Liaocheng University, Liaocheng 252000, China; jerryinkorea@gmail.com (D.L.); hyr018@163.com (Y.H.); 2 College of Architecture and Engineering, Liaocheng University, Liaocheng 252000, China

**Keywords:** urban rail transit, long-term passenger flow forecasting, complex networks, Informer model, time series analysis

## Abstract

With the continuous growth of urbanization, passenger flow in urban rail transit systems is steadily increasing, making accurate long-term forecasting essential for optimizing operational scheduling and enhancing service quality. However, passenger flow forecasting becomes increasingly complex due to the intricate structure of rail transit networks and external factors such as seasonal variations. To address these challenges, this paper introduces an optimized Informer model for long-term forecasting that incorporates the influences of other stations based on complex network theory. Compared to the ARIMA, LSTM, and Transformer models, this optimized Informer model excels in processing large-scale complex transit data, particularly in terms of long-term forecasting accuracy and capturing network dependencies. The results demonstrate that this forecasting approach, which integrates complex network theory with the Informer model, significantly improves the accuracy and efficiency of long-term passenger flow predictions, providing robust decision support for urban rail transit planning and management.

## 1. Introduction

As a critical component of modern urban infrastructure, urban rail transit systems play a significant role in alleviating traffic congestion, promoting environmental sustainability, and advancing urbanization. With the acceleration of urbanization, the passenger flow carried by these systems continues to increase, making accurate long-term passenger flow forecasting essential for ensuring efficient operation and informed planning [1]. Accurate forecasting enables optimized resource allocation, improved operational efficiency, enhanced passenger service quality, and strengthened system responses to emergencies.

Despite some progress in passenger flow forecasting for urban rail transit, significant limitations remain. Traditional statistical methods, such as ARIMA models, exhibit advantages in capturing linear trends in time series data but struggle to handle nonlinear variations and dynamic dependencies in complex networks, particularly when forecasting over long periods [2]. Deep learning models like LSTM and Transformer have shown superior performance in handling complex time series forecasting tasks; however, these models typically rely heavily on large amounts of high-quality training data and are sensitive to hyperparameter tuning, which may lead to unstable results [3].

Current research primarily focuses on forecasting passenger flow for individual stations or specific regions, with limited emphasis on the complex spatial dependencies across transit networks [4,5]. For instance, fluctuations in passenger flow at transfer hubs or high-traffic stations often create cascading effects on other stations. These space–time dependencies are not fully captured by existing forecasting methods [6]. While some approaches attempt to incorporate complex network theory to address these spatial interactions, challenges such as computational complexity and resource consumption remain unresolved [7]. These intricate interactions render passenger flow forecasting highly challenging, and traditional statistical methods often fail to account for dynamic changes and interdependencies within network components [8].

Based on this context, this study clearly articulates the following research questions: (1) How can nonlinear and dynamic dependencies within urban rail transit systems be effectively captured? (2) How can the accuracy of long-term passenger flow forecasting be improved in the context of multiple stations? Despite advancements in urban rail transit passenger flow forecasting, several limitations persist. Traditional models like ARIMA are effective in capturing linear trends but fail to handle nonlinear patterns and complex spatial dependencies within transit networks, especially for long-term predictions [9]. Deep learning models, such as LSTM and Transformer, improve accuracy but face challenges in computational efficiency and scalability when applied to intricate, real-time transit networks, making them less practical for large-scale dynamic systems [10].

To address the limitations of traditional forecasting methods in capturing the intricate dependencies within urban rail transit systems, this study aims to propose a long-term passenger flow forecasting algorithm that integrates complex network theory with the Informer model. Initially, models such as ARIMA, LSTM, and Transformer are used as benchmarks to assess their effectiveness in trend detection and managing long-term dependencies. However, given the highly interconnected structure and high-dimensional nature of urban rail systems, the focus shifts to leveraging the Informer model, which offers advantages in handling long-sequence data.

This study employs complex network theory to model the interdependencies among stations, capturing the intricate relationships that govern passenger flows across the system. The Informer model’s sparse self-attention mechanism enables the efficient processing of large-scale, high-dimensional data, significantly reducing computational complexity while preserving essential temporal and spatial information. Additionally, its temporal distillation technique enhances the model’s capacity to extract critical features, making it particularly well-suited for passenger flow forecasting in transit networks influenced by multiple interacting factors.

This study applies network science methodologies to quantify station-to-station influences by analyzing key factors such as connectivity and centrality. Through the application of complex network theory, it evaluates how these factors shape passenger flow patterns and their broader impact on the system. This approach enhances the accuracy of single-station forecasting while providing deeper insights into inter-station dynamics. The model’s performance is validated using real-world data, demonstrating its robustness and practical applicability for urban rail transit operations.

## 2. Related Research

### 2.1. Long-Term Passenger Flow Forecasting

The rapid expansion of urban rail transit systems in China, driven by increasing passenger demand and a growing emphasis on low-carbon urban operations, has led many cities to shift from the construction phase to a stage of stable development. In this critical transition, enhancing service quality and operational efficiency has emerged as a key objective [7]. Accurate passenger flow forecasting, encompassing both long-term and short-term predictions, is essential to achieve this goal. Long-term forecasting, in particular, plays a vital role in understanding how urban development influences transportation demand over time, necessitating in-depth analysis of factors such as population density and urban mobility [11].

Short-term forecasting typically predicts passenger flow within shorter time frames—such as the next 15 or 30 min—enabling real-time adjustments to transport supply based on immediate demand [12]. Conversely, long-term forecasting aims to predict travel demand over extended periods, such as one day or one week. Various methodologies have been employed for long-term passenger flow forecasting in urban rail systems, including statistical methods, regression analyses, classical time series models, deep learning techniques, and integrated models [13]. Statistical methods often encompass Bayesian techniques and kernel density estimation, while regression approaches range from linear to nonlinear methods, including polynomial regression, support vector regression, and decision tree regression. Classical time series models, notably the ARIMA model and its variants, remain prevalent. On the other hand, deep learning techniques, such as recurrent neural networks (RNNs), Long Short-Term Memory (LSTM), convolutional neural networks (CNNs), and Transformer models, have gained significant attention for their capacity to handle complex datasets [14].

### 2.2. Advancements in Forecasting Models

The ARIMA model has successfully been applied in various fields, including the prediction of COVID-19 spread, where it achieved high accuracy through parameter optimization [15]. Additionally, hybrid models that integrate ARIMA with artificial neural networks (ANNs) have demonstrated considerable improvements in forecasting performance, often outperforming ARIMA when used in isolation [16]. However, while ARIMA excels in analyzing time-dependent data, its performance diminishes when confronted with datasets exhibiting strong seasonality or nonlinear patterns [17].

In response to the limitations of traditional models, Long Short-Term Memory (LSTM) networks have emerged as effective tools for long-term time series forecasting. Introduced to mitigate issues such as vanishing gradients found in traditional RNNs, LSTMs have shown remarkable efficacy in various applications, including the analysis of long-term monitoring data, where they improve predictive performance while minimizing computational time and resource consumption [18,19]. In public transportation contexts, LSTM models excel in capturing long-term dependencies and managing variations in passenger flow, such as those caused by holidays or emergencies [20].

The introduction of the Transformer model in 2017 marked a significant leap in sequence-to-sequence tasks, such as machine translation [21]. Its core innovation lies in the self-attention mechanism, which facilitates the parallel processing of entire sequences, thereby reducing training time while simultaneously capturing relationships between all elements in a sequence [22]. This feature makes Transformers particularly adept at identifying long-term trends and periodic patterns in passenger flow. Advanced frameworks like the Meta Graph Transformer have been developed to capture temporal and spatial heterogeneity in Intelligent Transportation Systems (ITSs), further enhancing forecasting accuracy [23,24].

### 2.3. Challenges and Research Opportunities

Despite the advantages of complex network theory in modeling traffic flow within urban rail transit systems, several challenges persist [25]. One of the primary difficulties is the computational complexity involved in analyzing vast datasets from multiple stations over extended periods [26]. Large-scale networks typically consist of numerous nodes (stations) and edges (routes), making it computationally intensive to simulate passenger flows and predict dynamic changes within the system [27,28]. Additionally, optimizing multivariate data that incorporate factors such as station connectivity, passenger demand, and external influences like weather introduces further complexity to the model.

To address these challenges, researchers have explored various techniques, including the application of efficient algorithms for network decomposition and modularity maximization, along with advanced data handling methods such as dimensionality reduction and graph neural networks (GNNs). These techniques are designed to alleviate computational demands while retaining essential network characteristics [29]. Nonetheless, optimizing these approaches for real-time forecasting in transit systems remains an area requiring significant research. It is crucial to process dynamic, multivariate data streams efficiently and accurately [30].

In conclusion, multivariate forecasting methods, particularly those incorporating external factors like weather and holidays, have proven effective in enhancing the accuracy and reliability of long-term passenger flow predictions in rail transit systems [31]. While deep learning techniques such as LSTM have gained widespread application, the potential of models like the Informer—especially in addressing the complexities posed by intricate networks—remains underexplored. Further investigation into the Informer’s capabilities to enhance passenger flow prediction in urban rail systems is essential to fully harness its advantages and applicability [6,32].

## 3. Methodology

### 3.1. Overview of Informer

**Figure 1:**
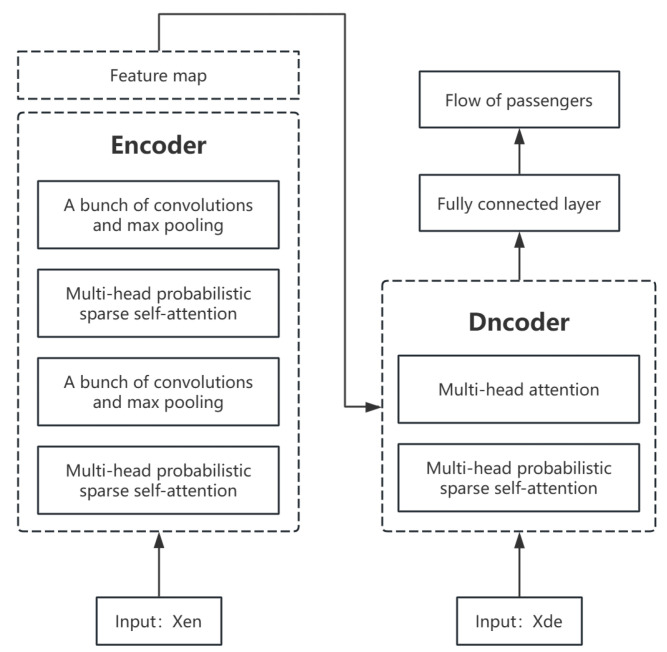
Informer model structure.

The Informer model’s architecture consists of two principal components: the encoder and the decoder. Each component is further subdivided into three distinct layers: a location embedding layer, a multi-head probabilistic sparse self-attention layer, and a convolutional distillation layer [33]. The Informer model processing workflow follows a structured sequence designed for efficient long-sequence time series forecasting (Figure 1).

First, the input data are passed through a temporal encoding layer, which embeds time-related and positional information to retain the order and relevance of the data. This step ensures that the model can recognize temporal patterns critical for accurate forecasting.

Next, deep feature extraction is performed through a network structure that incorporates a multi-head probabilistic sparse self-attention mechanism. This layer is a key innovation in Informer, allowing it to focus on the most relevant time points in a long sequence while reducing computational complexity. The convolutional layer further refines the feature representation by capturing local temporal dependencies in the data.

Finally, the output of these layers is fed into a fully connected layer, which generates the final passenger flow predictions. This process efficiently handles the high dimensionality and long-range dependencies of the input data, making Informer particularly suitable for applications like public transportation forecasting, where data exhibit both temporal and spatial dependencies.

Informer employs a linear transformation of the sine–cosine function for position encoding, which is calculated as follows: for the dimension of even index *i*, the position encoding utilizes the sine function. In the case of dimensions with an odd index, the position encoding employs the cosine function, as illustrated in Equation (1).

(1) $$\begin{array}{c} PE_{\left(\mathrm{pos},2i\right)}=sin\left(\frac{pos}{{10,000}^{2i/d_{\mathrm{model}\;}}}\right)\\ PE_{\left(pos,2i+1\right)}=cos\left(\frac{pos}{{10,000}^{2i/d_{\mathrm{model}\;}}}\right) \end{array}$$

The Informer model is an improved model based on Transformer, which can effectively reduce the computational time complexity by introducing a probabilistic sparse self-attention mechanism. In addition, the model puts special emphasis on key time points to improve the speed of prediction by generative decoding [34]. Informer has a standard codec structure: in the encoder part, the model transforms the input sequence X=(x1,x2,...,xn) into the corresponding continuous sequence of hidden states $Z=\left(z1,z2,...,zn\right)$, and the decoder generates the entire predicted sequence Y=(y1,...,yn) in one go based on these hidden states.

The encoder comprises six identical layers, each comprising two sublayers. The first of these is a multi-head self-attention mechanism, while the second is a fully connected feed-forward network. In this context, each sublayer is represented as a function, Sublayer(x) which is responsible for processing the input x and outputting the processed result.

The decoder is also composed of six identical layers, each with an output dimension of 512. Similarly, the decoder comprises two fundamental sublayers in each layer: a multi-head self-attention mechanism and a fully connected feed-forward network. The decoder differs from the encoder in that it incorporates a third sublayer, which is utilized for the multi-head attention mechanism on the encoder output. The outputs of each sublayer are connected via residuals and subsequently subjected to layer normalization.

The conventional approach to implementing the probabilistic sparse self-attention mechanism is illustrated in the Equation (2):

(2) $$Z=\;\mathrm{Attention}\;\left(Q,K,V\right)=softmax\left(\frac{QK^{T}}{\sqrt{d_{k}}}\right)V$$

In this context, the probabilistic sparse self-attention mechanism can be expressed as follows:

(3) $$A\left(Q,K,V\right)=softmax\left(\frac{\bar{Q}K^{T}}{\sqrt{d}}\right)$$

The multi-head probabilistic sparse self-attention mechanism may cause redundancy in feature mapping in the encoder. To address this issue, the Informer model employs a distillation mechanism, which helps to extract more key features in subsequent layers by increasing the emphasis on key node data features. The distillation mechanism uses a one-dimensional convolution and max pooling method to down sample the time dimension of the input, thereby reducing the sequence lengths of Q(Queries) and K(Keys). Specifically, the transformation from layer *d* to layer *d* + 1 is realized in this way.

(4) $$X_{d+1}^{t}=MaxPool\left(ELU\left(\mathrm{Convld}\left({\left[X_{d}^{t}\right]}_{AB}\right)\right)\right)$$

In Equation (4), ${\left[X_{d}^{t}\right]}_{AB}$ represents the key distinguishing operation between the multi-head probabilistic sparse self-attention mechanism and the conventional self-attention mechanism. Next, a one-dimensional convolution operation *Convld* of the temporal data is performed and the *ELU* activation function is used. After this step, the input sequences are significantly reduced by a maximum pooling layer (*MaxPool*) to reduce the computational resources and memory usage to improve the prediction efficiency.

The traditional decoder in the Transformer model generates the output incrementally, predicting data only one time step at a time. In contrast, the Informer model uses a generative decoder that generates the entire output sequence at once. Such a decoder consists of two decoder layers. inside each decoder layer, a multi-head probabilistic sparse self-attention mechanism with masking and a multi-head attention mechanism is included. The multi-headed probabilistic sparse self-attention mechanism with mask incorporates a mask to prevent the model from accessing data at future time points to avoid the autoregressive problem.

In the Informer model, the input sequence to the decoder consists of two parts: one part is the sequence to be predicted, and the other part is a piece of known sequence before the point in time to be predicted. For example, in the scenario of predicting the passenger flow of each train for the next 14 days, the input to the decoder includes the passenger flow of each train for the 14 days before the point in time to be predicted as a known sequence, as well as the passenger flow data of each train for the previous week is also incorporated as part of the input. Thus, the input to the decoder *Xfeed*_*deco* *der* is described as Equation (5):

(5) $$X_{\mathrm{feed}\_\mathrm{decoder}}=\;\mathrm{concat}\;\left(X_{\mathrm{token}\;},X_{\mathrm{placeholder}}\right)\in R^{\left(L_{\mathrm{token}}+L_{y}\right)\times d}$$

In this expression, *X*_*token*_ represents a piece of known sequence before the sequence to be predicted and is used as a label; *X*_*place*_*_h_*_*older*_ is the sequence to be predicted and all its elements are filled with zeros; *L*_*token*_ denotes the length of the historically known sequence; and *L*_*y*_ is the length of the sequence to be predicted. After processing through the decoder, each location to be predicted corresponds to a vector, and these vectors are subsequently fed into the fully connected layer to obtain the final passenger flow prediction results.

### 3.2. Optimization of Informer

In rail transit systems, passenger flow prediction is crucial for optimizing operational management and rational resource allocation. Although traditional prediction models such as ARIMA and LSTM perform well in short-term forecasting, they are often structurally limited in dealing with long-term time series data, making it difficult to effectively capture remote dependencies and complex temporal dynamics. To address this issue, researchers have introduced the Informer model, which primarily leverages historical data related to the forecasting target. However, historical data from individual stations often fail to fully reflect the complex dynamic passenger flow patterns in rail transit systems.

**Figure 2:**
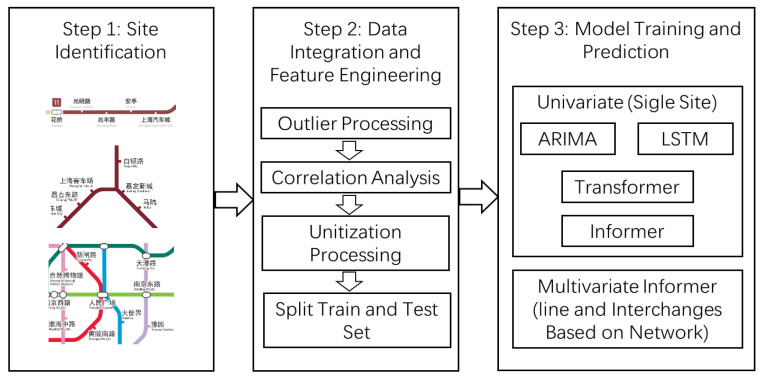
The research framework of Informer’s long-term passenger flow prediction is based on a complex network.

To further enhance the accuracy and practicality of predictions, this study proposes an extended long-term passenger flow prediction framework based on complex networks and the Informer model. This framework not only utilizes the historical data from the target station but also integrates multi-source data from other related stations, thereby improving the model’s ability to capture spatial correlations and internal interactions within the system, which significantly enhances prediction performance. The specific research framework is illustrated in Figure 2, showing how the model can be applied to long-term passenger flow prediction in complex rail transit networks. By incorporating a model architecture diagram, the integration of complex networks with the Informer model can be visualized. The encoder of the Informer model processes time series data, while the complex network module models inter-station relationships to enhance predictions. This architecture enables the model to handle both temporal sequences and capture spatial dependencies between stations.

- Related site identification: The first step involves selecting stations based on network characteristics. A single station without other variable influences was chosen as the baseline, with the ARIMA, LSTM, Transformer, and Informer models used as prediction benchmarks. Additionally, stations from the same line were selected as feature inputs. Furthermore, three surrounding stations with a centrality degree of 3 were selected as additional feature inputs, and the model was finally validated using the Informer model.
- Data integration and feature engineering: After identifying the relevant stations, the historical passenger flow data from these stations are collected and integrated with the data from the target station. During the feature engineering phase, data from midnight to 5 a.m. are excluded, as the stations only operate from 5 a.m. onwards. Given that the data are collected in real-time, they are aggregated on an hourly basis to ensure consistency across models. In particular, for the ARIMA model, time window segmentation and the creation of lagged features are performed to uncover the underlying dynamic patterns within the time series data.
- Model training and prediction: By integrating data from multiple related stations, a more comprehensive and representative dataset is provided for all models. In the univariate (single-station) models, the ARIMA, LSTM, Transformer, and Informer models are employed to identify the predictive advantages of the Informer model for individual stations. Subsequently, the proposed network-based multi-station model further optimizes the Informer model by incorporating line-based and network-based data. Through model training, the Informer model not only learns the temporal characteristics of the target station but also captures the interactions and influences between stations. This approach enables the Informer model to better understand how multiple stations collectively affect passenger flow dynamics, leading to more accurate long-term passenger flow predictions.
- Model validation and tuning: During the model validation phase, the effectiveness of the model is assessed by comparing the actual passenger flow data from the final week’s validation dataset with the predicted results. Additionally, the performance differences among the various models are analyzed to evaluate the effectiveness of the optimized Informer model.

### 3.3. Evaluation Metrics

In this study, the performance of the model is evaluated using four indicators: Mean Absolute Error (MAE), Mean Squared Error (MSE), Root Mean Squared Error (RMSE), and Mean Absolute Percentage Error (MAPE).

MAE directly reflects the absolute error between the predicted and actual values, measuring the absolute level of prediction accuracy. MSE emphasizes the impact of larger errors by squaring each error, making it sensitive to large deviations and ensuring positive values. This characteristic makes MSE particularly useful for optimization algorithms. RMSE, as the square root of MSE, provides an interpretable measure of error in the same units as the original data, making it easier to assess the model’s overall prediction accuracy.

MAPE, on the other hand, measures the percentage error between the predicted and actual values, providing a normalized metric that expresses the error as a percentage. It is especially useful for comparing performance across datasets with different scales or units, as it standardizes the error relative to the actual values. A lower MAPE indicates a higher prediction accuracy, with values closer to zero representing more accurate predictions.

The formulae for the indicators are shown below:

(6) $$MAE=\frac{1}{n}\sum_{i=1}^{n}\;\left|y_{i}-{\hat{y}}_{i}\right|$$

where $y_{i}$ is the actual value, ${\hat{y}}_{i}$ is the predicted value, and n is the total number of samples.

(7) $$MSE=\frac{1}{n}\sum_{i=1}^{n}\;{\left(y_{i}-{\hat{y}}_{i}\right)}^{2}$$

(8) $$RMSE=\sqrt{\frac{1}{n}\sum_{i=1}^{n}{\left(y_{i}-{\hat{y}}_{i}\right)}^{2}}$$

(9) $$MAPE=\frac{100\%}{n}\sum_{i=1}^{n}\;\left|\frac{y_{i}-{\hat{y}}_{i}}{y_{i}}\right|$$

## 4. Experimentation and Analysis

### 4.1. Data Selection and Feature Engineering

#### 4.1.1. Data Selection

The dataset used in this study is derived from the 2015 SODA competition and includes card swiping records from April 2015 provided by the Shanghai Public Transportation Company (SPTC). It encompasses detailed records of card-swiping events across the bus and metro systems, offering a valuable empirical foundation for research in traffic pattern recognition and passenger flow prediction. The main fields of the dataset are as follows:

- Card ID: This is a unique identifier assigned to each One Card, allowing for the distinct identification of individual passengers within the transit system.
- Line and station information: This captures detailed data regarding the specific subway line and station where each card swipe transaction occurs, offering insight into passenger movements across the transit network.
- Fee: This represents the fare charged to passengers for each trip, based on variables such as distance traveled or time spent in transit.
- Discounts/offers: This denotes any promotional discounts or offers applied to the fare during bus or subway rides, reflecting variations in pricing based on rider-specific incentives.
- Swipe timestamp: This logs the precise time of each card swipe, enabling detailed temporal analysis of passenger flow patterns and travel behavior across different periods. To facilitate a detailed analysis of ridership patterns across various time intervals, an additional “hour” variable was introduced into the model.

This variable enables the segmentation of passenger data by specific periods, allowing for a more granular exploration of ridership trends. In the context of subway systems, passengers typically do not incur a fare upon entry (i.e., inbound card swipes have a cost of zero). Instead, fares are charged at exit points, where the total cost is calculated based on either the distance traveled or the duration of the journey. By leveraging this fare structure, this study accurately pairs entry and exit records, enabling the precise identification of passengers’ origin and destination stations. This approach provides robust data for conducting passenger flow analyses and enhances the overall accuracy of ridership modeling.

#### 4.1.2. Data Feature Engineering

For the passenger flow data at each station, derived from metro swipe card records, effective feature engineering is crucial for accurate prediction and analysis. By performing an in-depth analysis of the raw data, more representative features can be extracted, such as differences in flow between peak and off-peak hours, weekday versus holiday flow patterns, and the time intervals between entry and exit at stations. Additionally, incorporating external factors such as weather changes and major events can further enhance the model’s ability to capture dynamic changes in passenger flow. The extraction and selection of these features not only improve the model’s accuracy in predicting future passenger flow trends but also provide valuable insights to support the optimization and management of rail transit systems.

**Figure 3:**
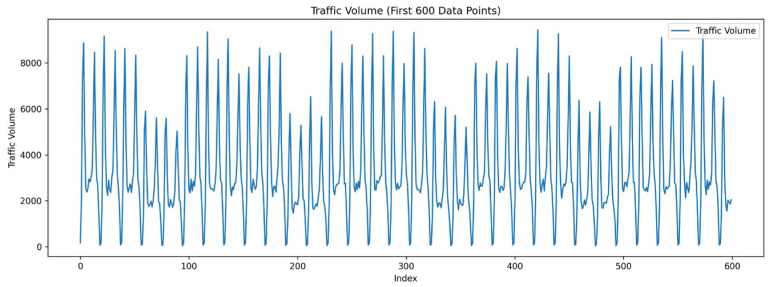
Characteristics of passenger flow data at one sample station.

- Data cleaning: Data cleaning is an essential preliminary step to ensure data quality and consistency. Initially, missing values are addressed using a deletion strategy to avoid biasing the analysis with incomplete data. Additionally, the detection and treatment of outliers are crucial to maintaining the accuracy and reliability of the data by identifying and removing unreasonable data points. To facilitate the extraction of temporal features and perform temporal analysis, type conversion is required, particularly converting timestamps to datetime objects.
- Data transformation: Rail transit passenger flow data exhibit distinct and significant characteristics influenced by various factors, including time (hourly, daily, weekly), type of day (weekday versus weekend or holiday), and seasonal variations. Passenger flows in rail transit show a pronounced daily cyclicality, as illustrated in Figure 3. Typically, passenger flow increases gradually from early morning, peaks in the morning, drops slightly during midday, rises again in the afternoon, reaches another peak in the evening, and then gradually decreases until late at night. This pattern is closely aligned with people’s daily work and study schedules. On weekdays, patronage is generally higher compared to weekends, particularly during the morning and evening peak periods, when commuting demands are at their highest. In contrast, weekend traffic tends to be lower and more evenly distributed throughout the day, likely due to the more flexible nature of weekend activities. The rail system usually operates from 5 a.m. until late at night, leading to minimal or zero patronage during the early hours before 5:00 a.m.
- Feature extraction: Once data cleaning is complete, creating new features from existing data is vital for enhancing the model’s predictive power. This study emphasizes two key aspects of feature extraction: temporal features and historical data features. Temporal feature extraction involves deriving information such as year, month, day, and hour from timestamps, and distinguishing between peak and off-peak hours to provide the model with detailed time-based information. Historical data features involve using past passenger flow data (e.g., passenger flow at the same time on the previous day or week) as input features, which helps the model capture cyclical patterns in passenger flow changes more effectively.
- Feature scaling: Scaling of the extracted features is necessary to ensure the stability and efficiency of the model training process. In this study, the Min-Max normalization method is used to scale feature values to the range of 0 to 1. This normalization process helps prevent model instability due to large differences in feature values, accelerates model convergence, and improves prediction accuracy.

To enhance prediction accuracy, data from 12:00 a.m. to 5:00 a.m. were excluded from the analysis to mitigate the impact of low passenger flow during this period on the model. To validate the accuracy of each model, this study used the last 133 time points (representing one week of data) as the validation set for the Transformer and Informer models. Meanwhile, for all other models, the remaining data (excluding the final week) were split into training and testing sets in an 8:2 ratio to ensure robustness and comparability of the results.

### 4.2. Model Building

In the complex network-based Informer model for long-term passenger flow prediction in rail transit, this study has been expanded across multiple dimensions, including model-specific implementation, performance evaluation, parameter tuning, and comparative analysis with other models.

In the selection of forecasting models, this study compares several architectures to identify the most suitable solution for rail transit passenger flow forecasting. The ARIMA, LSTM, and Transformer models are widely used in time series processing. The ARIMA model is commonly employed to analyze and predict data series with time dependence, where autoregressive coefficients and smoothing coefficients are determined by ACF and PACF analyses. LSTM, a variant of the recurrent neural network, excels in processing time series data, particularly in handling time-dependent tasks, and effectively mitigates long-term dependency issues through its gating mechanism.

The Transformer model captures dependencies between different positions in the sequence through the attention mechanism, making it particularly suitable for processing long sequences and offering higher computational efficiency compared to traditional RNN models. Informer, an enhanced version of Transformer, is optimized for long-term sequence prediction and employs a more efficient attention mechanism (e.g., probabilistic sparse attention) to reduce computational resource consumption and improve prediction performance. The specific parameter settings for these models are detailed in Table 1.

By comparing the performance of these models, the Informer model was finally selected for long-term passenger flow forecasting in rail transit in this study, which verified its superiority in handling long-time series data.

### 4.3. Analysis of Results

#### 4.3.1. Univariate Informer Outcome Analysis

**Figure 4:**
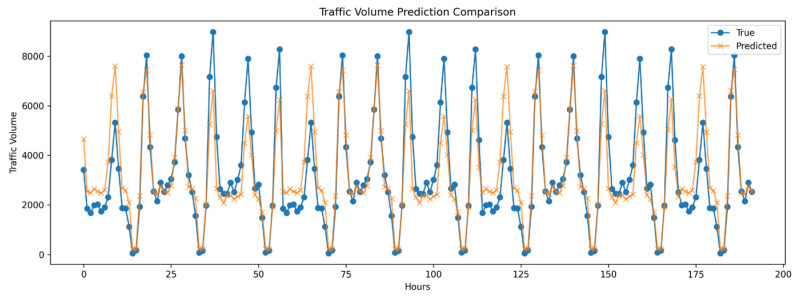
Univariate Informer model results for long-term traffic forecasting.

The Informer model is specifically designed to address the challenges associated with long-term time series prediction, excelling particularly in handling ultra-long sequence data. While based on the Transformer architecture, Informer further enhances the efficiency and effectiveness of processing long sequences through the introduction of a probabilistic sparse attention mechanism. The primary advantage of Informer lies in its efficient attention mechanism, which improves the model’s accuracy and reduces computational resource demands when capturing long-distance dependencies. The results of applying the Informer model for single-site prediction are presented in Figure 4, showcasing its superior performance in long-term time series forecasting.

#### 4.3.2. Analysis of Multivariate Informer Results Based on Complex Networks

The integration of complex network theory with the Informer model for long-term, multi-station passenger flow prediction in rail transit systems provides notable advantages. Complex networks offer a robust framework for simulating and modeling the interdependencies among stations within the transit system, enabling a more nuanced and accurate understanding of passenger flow dynamics. By representing the interactions between different stations, complex network theory facilitates a comprehensive analysis of how changes at one station can affect the entire system.

Furthermore, the Informer model’s multi-scale temporal processing significantly enhances its predictive capacity for capturing long-term passenger flow fluctuations. Given that passenger flow in rail transit systems is often influenced by the interconnectivity between stations, the Informer model is particularly adept at recognizing and modeling these intricate correlation patterns over multiple time scales. This capability allows it to predict not only short-term variations but also long-term trends in passenger behavior.

**Figure 5:**
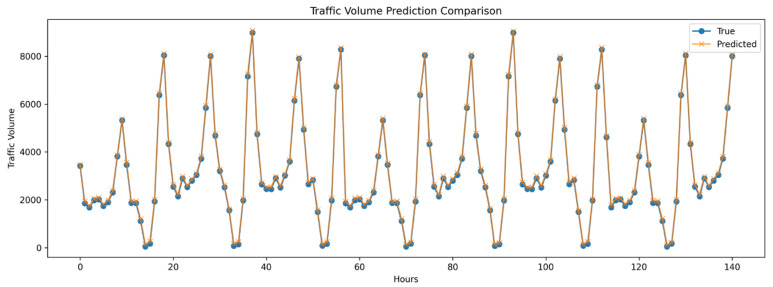
Long-term passenger flow prediction across 10 stations using the Informer model.

**Figure 6:**
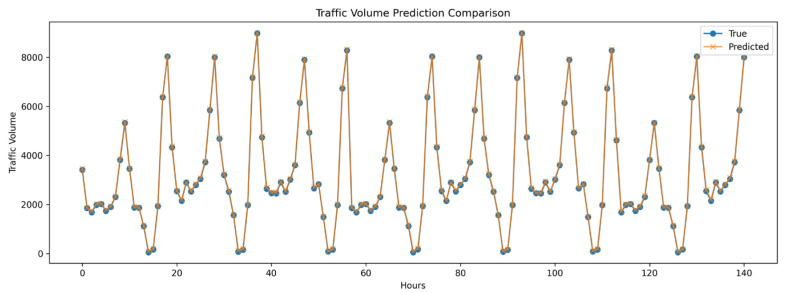
Long-term passenger flow prediction for transfer stations using the Informer model. (Centrality degree of 3; 9 stations.)

Overall, the integration of complex network theory and the Informer model for long-term, multi-station passenger flow forecasting significantly improves prediction accuracy while enhancing system management efficiency. This approach provides practical value for sustainable development planning, as it enables rail transit operators to better understand and respond to dynamic passenger flow patterns, improving both service quality and operational effectiveness. The predictive outcomes, particularly those focused on online-based and interchange station flow analysis (as depicted in Figure 5 and Figure 6), further validate the effectiveness of the Informer model in this context, underscoring its superiority over traditional methods.

#### 4.3.3. Evaluation Results

Table 2 provides a comprehensive comparison of various models used for long-term passenger flow prediction in rail transit systems, focusing on four critical performance metrics: Mean Squared Error (MSE), Root Mean Squared Error (RMSE), Mean Absolute Error (MAE), and Mean Absolute Percentage Error (MAPE). These metrics offer insight into the accuracy and robustness of each model, where lower values indicate better performance.

The ARIMA model, as a traditional time series forecasting method, performs the weakest, displaying notably high error values across all metrics. This indicates its limitations in capturing the complex and dynamic passenger flow patterns in rail transit systems. Although ARIMA provides a baseline, its high MSE, RMSE, MAE, and MAPE values reflect its shortcomings in modeling long-term dependencies and nonlinear interactions. Additionally, while the ARIMA model has relatively low computational complexity, typically with linear time complexity $O\left(n\right)$, its efficiency advantage cannot compensate for its poor prediction performance, especially in handling complex nonlinear patterns.

The LSTM model, a deep learning technique for managing long-term dependencies, shows significant improvement over ARIMA. Its metrics see considerable reduction, demonstrating LSTM’s strength in capturing long-term temporal patterns. However, LSTM’s time complexity is $O\left(n^{2}\right)$, which leads to a significant increase in computational resources and time, especially when processing long sequences. Compared to more advanced models, LSTM is not optimal in terms of computational efficiency or performance, and it faces limitations in modeling complex interactions across multiple stations.

The Transformer model performs even better, achieving lower error rates than LSTM. By using a self-attention mechanism to process entire sequences simultaneously, it effectively models the complex and dynamic behaviors inherent in passenger flow data. The Transformer model’s time complexity is $O\left(n\cdot \log n\right)$, offering greater efficiency in processing long sequences compared to LSTM. Its success in reducing error values further emphasizes its ability to handle both temporal and spatial dependencies.

The Informer model, particularly when applied to specific contexts such as individual lines and interchange stations, delivers the best performance. In its univariate form, Informer performs competitively, though slightly behind Transformer. However, when expanded to a multi-station approach, especially focusing on lines and interchange stations, the Informer model significantly outperforms all other models. By introducing a sparse self-attention mechanism, Informer reduces time complexity to $O\left(\log n\right)$, greatly decreasing computational time and resource consumption while maintaining high prediction performance. Its exceptional ability to capture complex, multi-scale temporal patterns and spatial interactions is reflected in the lowest MSE, RMSE, MAE, and MAPE values. Notably, the model performs best when applied to interchange stations, where passenger flow patterns are most intricate due to the high interconnectivity of the network.

**Figure 7:**
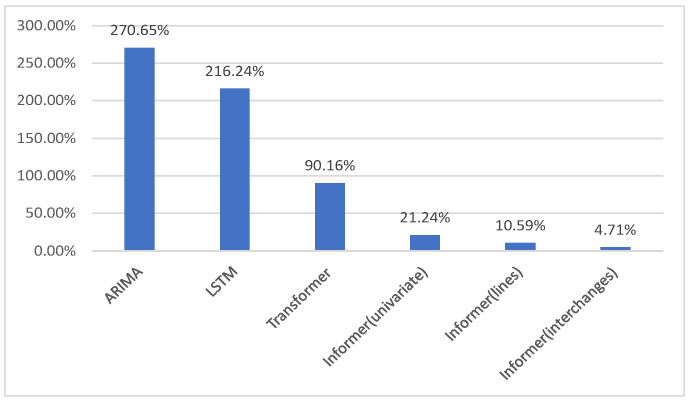
The comparison of MAPE values across different models.

Among the MSE, RMSE, MAE, and MAPE metrics, MAPE is a commonly used metric for evaluating the performance of forecasting models. It measures the deviation between the predicted and actual values as a percentage, with lower values indicating higher prediction accuracy. Figure 7 shows the comparison of MAPE values across different models.

Both the ARIMA and LSTM models show relatively poor performance in this forecasting task, with MAPE values of 270.65% and 216.24%, respectively. ARIMA, as a traditional time series model, struggles to capture complex, nonlinear patterns in the data, leading to a high error. While LSTM, a deep learning model designed for time series, performs slightly better, its error remains significantly high, suggesting that it also has limitations in this context, particularly compared to more advanced models. The Transformer model significantly improves prediction accuracy with a MAPE of 90.16%, outperforming ARIMA and LSTM by effectively capturing long-range dependencies, though there is still potential for further refinement.

The Informer model, an enhanced version of the Transformer, shows progressively improved performance across different data types. For univariate stations, it reduces the MAPE to 21.24%, for line stations to 10.59%, and for multiple stations to an impressive 4.71%. This demonstrates the model’s superior ability to handle complex and multivariate data, significantly outperforming other models in prediction accuracy.

In summary, the results highlight the superiority of the Informer model, especially in complex, multi-station, and long-term forecasting tasks. By effectively integrating both spatial and temporal dimensions, the Informer model significantly enhances prediction accuracy and operational efficiency, while demonstrating clear advantages in terms of time complexity and computational resources, making it the most suitable solution for rail transit management.

## 5. Conclusions

In this study, we propose a long-term passenger flow forecasting algorithm for urban rail transit that integrates complex network theory with the Informer model. This approach aims to address the limitations of traditional forecasting methods by capturing the complex dependencies present within urban rail transit systems.

We utilized models such as ARIMA, LSTM, and Transformer as benchmarks to evaluate their effectiveness in trend detection and the management of long-term dependencies. However, given the highly interconnected structure and high-dimensional nature of urban rail systems, we shifted our focus to the Informer model, which demonstrates exceptional performance in handling long-sequence data.

The application of complex network theory in this study allows for the modeling of interdependencies among stations, thereby capturing the intricate relationships that govern passenger flows. The sparse self-attention mechanism of the Informer model efficiently processes large-scale, high-dimensional data, significantly reducing computational complexity while preserving essential temporal and spatial information. This enhancement in predictive capability makes the model particularly suitable for forecasting passenger flow influenced by multiple interacting factors.

Furthermore, by employing network science methodologies, this research quantifies the influences between stations by analyzing key factors such as connectivity and centrality. This evaluation not only enhances the accuracy of single-station forecasts but also provides deeper insights into inter-station dynamics. The validation of our model using real-world data demonstrates its robustness and practical applicability within urban rail transit operations.

Ultimately, this study underscores that effective long-term passenger flow forecasting can significantly improve operational efficiency and service quality in urban rail systems. The integration of complex network theory with advanced forecasting models not only addresses current limitations but also establishes a framework for future research and practical applications. By achieving these objectives, we contribute to the optimization of urban mobility and the enhancement of the sustainability of transportation systems.

This research introduces a significant theoretical contribution by combining complex network theory with the Informer model for long-term passenger flow forecasting. The integration of these two frameworks offers a new perspective on how interdependencies between stations influence passenger flows, a challenge not fully addressed by existing models. The theoretical foundation provided by complex network theory enables modeling of station connectivity, transmission pathways, and clustering effects within the transportation network. By analyzing topological properties such as degree distribution, clustering coefficients, and modularity, we reveal the core influence of key stations on overall passenger flow patterns. For example, major transfer hubs can act as critical nodes in the network, where fluctuations may trigger cascading effects throughout the system. This network-based perspective not only enhances the accuracy of single-station passenger flow predictions but also deepens our understanding of how interactions between stations influence the system as a whole.

The practical contribution of this research lies in its ability to enhance the accuracy and efficiency of passenger flow predictions in large-scale urban rail systems. By addressing limitations in traditional models such as ARIMA and LSTM, this study provides a more reliable tool for transit operators to optimize resource allocation, enhance service quality, and strengthen system resilience against fluctuations. The adoption of the Informer model, particularly with its sparse self-attention mechanism, allows for more efficient processing of high-dimensional data, leading to faster and more scalable forecasting that can be implemented in real-time transit management systems.

The experimental results demonstrate that the complex network-based Informer model substantially outperforms traditional models in predicting passenger flow across high-dimensional datasets and complex station networks. These traditional models often struggle with multi-dimensional spatial dependencies, whereas our approach compensates for these limitations, significantly improving forecasting outcomes. Moreover, the proposed model provides strong decision-making support for optimizing resource allocation, enhancing emergency response, and planning for long-term operations in real-world urban transit systems.

Another major contribution is the focus on multi-station networks rather than individual stations, acknowledging the cascading effects of passenger flow changes across interconnected systems. This research offers a deeper understanding of how transfer hubs and high-traffic stations impact the entire network, providing transit planners with comprehensive insights for long-term system planning, emergency management, and peak-hour optimization.

The use of real-world data, such as that from the Shanghai Public Transportation Company, adds to this paper’s practical relevance. The validated results underscore the model’s robustness and applicability, providing actionable insights for cities and operators aiming to improve urban mobility. By leveraging real-world data, this study bridges the gap between theoretical research and practical application, making it a pivotal work in the fields of transportation planning, complex networks, and machine learning.

Despite the promising results of this study, several limitations remain. First, the data used in this research come from a specific time period and location (Shanghai Public Transportation Company), which may limit the generalizability of the findings. Future research should consider applying datasets from other regions and time periods to validate the model’s robustness and scalability in different urban rail transit systems.

Second, while this study utilized passenger flow data from stations highly correlated with the target station as model input features, this approach is more related to feature engineering rather than a full application of complex network theory. The model did not account for global and local metrics of complex networks, which somewhat limits the understanding of the overall dynamics of the transportation network. Future research could further explore relevant metrics in complex network theory, such as degree distribution, clustering coefficients, and modularity, to enhance the model’s ability to predict system-wide traffic patterns.

Additionally, future research could explore the integration of complex network theory with deep learning models, such as hybrid models that combine the strengths of Informer, LSTM, and Transformer, to develop more robust models capable of handling the increasing complexity and variability of urban transit systems. Enhancing real-time data processing capabilities, incorporating external factors (e.g., weather, economic activity), and expanding the model’s applicability across different regions would significantly increase its practical value and innovation. By addressing these areas, we aim to confirm the broader applicability of the model and ensure its effectiveness in diverse environments.

## Figures and Tables

**Table 1:** Neural network model parameters.

Hyperparameterization	LSTM	Transformer	Informer
Store	1	4	4
Number of hidden units	64	64	64
Learning rate	0.001	0.001	0.001
Activation function	Relu	Relu	Relu
Optimizer	Adam	Adam	Adam
Number of self-attention heads		4	4
Number of self-attention layers		4	4
Attention head size		16	64
Number of training rounds	200	200	200
Forecast days	3	3	3

**Table 2:** Results of model evaluation indicators.

Method	MSE	RMSE	MAE	MAPE
ARIMA	20,199,897	4494	3963	270.65%
LSTM	2,560,735	1600	1255	216.24%
Transformer	87,3204	934	644	90.16%
Informer (univariate)	96,0537	980	681	21.24%
Informer (lines)	5894	98	96	10.59%
Informer (interchanges)	1099	33	30	4.71%

## Data Availability

The data presented in this study are available from the corresponding author upon request. The data are not publicly available due to the inclusion of site geographic information.
